# Relationship between platelet-related parameters and new-onset atrial fibrillation after coronary bypass surgery

**DOI:** 10.1590/1806-9282.20240076

**Published:** 2024-05-20

**Authors:** Abdurrahman Demirel, Mesut Engin, Faruk Toktas, Kadir Kaan Özsin, Ahmet Kağan As, Ufuk Aydın, Yusuf Ata, Şenol Yavuz

**Affiliations:** 1 University of Health Sciences, Bursa Yuksek Ihtisas Training and Research Hospital, Department of Cardiovascular Surgery – Bursa, Turkey.

**Keywords:** Platelets, Inflammation, Coronary artery bypass surgery, Atrial fibrillation

## Abstract

**OBJECTIVE:**

Inflammation plays a key role in the pathogenesis of postoperative atrial fibrillation after coronary artery bypass graft surgery. In this study, we aimed to investigate the changes in mean platelet volume and platelet values during the spring and autumn seasons in patients who underwent isolated coronary artery bypass graft surgery and the possible effect of these occurrences on postoperative atrial fibrillation.

**METHODS:**

Consecutive patients who underwent elective isolated coronary bypass surgery at our clinic in the spring and autumn months, between August 2020 and July 2022, were retrospectively included in this study. Variables were evaluated according to the spring and autumn seasons. Patients who did not develop in-hospital postoperative atrial fibrillation were identified as Group 1, and those who did constituted Group 2.

**RESULTS:**

A total of 622 patients were included in the study. The patients were divided into two groups: those who were operated on in the spring (n=277, median age=62 years, male gender ratio=77.3%) and those who were operated on in the autumn (n=345, median age=61 years, male gender ratio=81.4%). There was no statistically significant difference between the patients operated on in both seasons in terms of age, gender, hypertension rates, and the frequency of chronic obstructive pulmonary disease. In multivariate analysis, being over 70 years old (OR: 1.934, 95% confidence interval (CI) 1.489–2.995, p<0.001), having a left ventricular ejection fraction below 30% (OR: 1.550, 95%CI 1.190–2.236, p=0.012), and having chronic obstructive pulmonary disease (OR: 1.663, 95%CI 1.339–2.191, p<0.001) were found to be independent predictors in predicting the development of postoperative atrial fibrillation.

**CONCLUSION:**

In this study, we first demonstrated that mean platelet volume and platelet mass index values were higher in patients in the autumn months. Additionally, for the first time in the literature, we showed that there is a significant relationship between platelet mass index value and the development of postoperative atrial fibrillation in patients who underwent isolated coronary artery bypass graft.

## INTRODUCTION

Coronary artery disease (CAD) is a significant atherosclerotic cardiovascular disease and its appropriate treatment is of vital importance. Although endovascular interventions are increasing today, coronary artery bypass graft (CABG) surgery remains foremost. Although these operations are performed with high success rates, some postoperative complications may occur^1^. One of the most critical complications is postoperative atrial fibrillation (PoAF), which is detected at a rate of 20–40% after CABG operations and is significant in that it causes heart failure, cerebrovascular events, and prolonged hospitalization^2^.

Inflammation plays a key role in the pathogenesis of PoAF. Platelets have an important place in the pathogenesis of inflammation, and mean platelet volume (MPV) is an important marker of platelet functions^3^. Various studies have demonstrated the effects of platelet-related parameters on PoAF after CABG operations^4^. It has also been shown that there may be changes in MPV values depending on the seasons^5^.

In this study, we aimed to investigate the changes in MPV and platelet values, during the spring and autumn seasons, in patients who underwent isolated CABG surgery and the possible effect of these occurrences on PoAF.

## METHODS

Consecutive patients who underwent elective isolated coronary bypass surgery at our clinic in the spring and autumn months, between August 2020 and July 2022, were retrospectively included in this study. Prior approval was obtained from the clinical research ethics committee of our hospital (2011-KAEK-25 2022/10-10). Demographic data (age, gender, chronic disease, etc.), preoperative blood values (hemoglobin, neutrophil, lymphocyte, platelet, MPV, urea, creatinine, etc.), operative characteristics (total perfusion time, cross-clamp time, etc.), and postoperative characteristics (PoAF development, etc.) of all patients during the perioperative period were recorded. After the exclusion criteria, a total of 622 consecutive patients were included in the study. Variables of the patients were evaluated according to spring and autumn seasons. Also, patients who did not develop in-hospital PoAF were identified as Group 1, and those who did constituted Group 2.

### Statistical analysis

The SPSS 21.0 (IBM Statistical Package for the Social Sciences Statistic Inc., version 21.0, Chicago, IL, USA) computer program was used to analyze the data. Means and standard deviations (SDs) were calculated using descriptive methods for continuous and ordinal data. Kolmogorov-Smirnov test and Shapiro-Wilk test were used for normality distribution. For normally distributed data, Student's t-test was used, and the data were shown as mean±SD. For data that did not comply with normal distribution, the Mann-Whitney U test was used and the data were expressed as mean (minimum–maximum). Frequency and percentage analysis was performed for nominal data, and the chi-square test was used to compare these data. Multivariate logistic regression analysis was used to analyze the predictors of PoAF. For correlation analysis, Spearman correlation analysis was used. A p-value of less than 0.05 was considered statistically significant.

## RESULTS

A total of 622 patients were included in the study. The patients were divided into two groups: those who were operated on in the spring (n=277, median age=62 years, male gender ratio=77.3%) and those who were operated on in the autumn (n=345, median age=61 years, male gender ratio=81.4%) (Table 1). There was no statistically significant difference between the patients operated on in both seasons in terms of age, gender, hypertension rates, diabetes mellitus, hyperlipidemia rates, and the frequency of chronic obstructive pulmonary disease (COPD). In addition, the ejection fractions, body mass indexes, white blood cell values, hematocrit values, and platelet counts of the patients operated on in both seasons were statistically similar. Only MPV and PMI values were statistically significantly higher in patients operated in the autumn (p<0.001 and p=0.005, respectively).

**Table 1:** Characteristics of patients according to spring and autumn seasons.

Variables	Spring (n=277)	Autumn (n=345)	p-value
Age (years)	62 (39–89)	61 (38–85)	0.342‡
Male gender, n (%)	214 (77.3%)	281 (81.4%)	0.197*
Hypertension, n (%)	189 (68.2%)	239 (69.3%)	0.780*
Diabetes mellitus, n (%)	87 (31.4%)	103 (29.9%)	0.676*
Hyperlipidemia, n (%)	109 (39.4%)	146 (42.3%)	0.454*
Smoking, n (%)	108 (39%)	124 (35.9%)	0.435*
COPD, n (%)	29 (10.5%)	41 (11.9%)	0.579*
Cerebrovascular event, n (%)	17 (6.1%)	32 (9.3%)	0.149*
BMI, (kg/m^2^)	27.3 (23–39.7)	26.6 (24–41.1)	0.491
LVEF (%)	50 (20–72)	50 (25–67)	0.114‡
SYNTAX score I	15 (9–41)	17 (7–39)	0.362‡
PoAF, n (%)	93 (33.6%)	110 (31.9%)	0.655
White blood cell (10^3^/μL)	8.5 (3.8–10.6)	8.4 (4.3–9.6)	0.635
Hematocrit (%)	40.8 (29–52.2)	39.6 (23–55)	0.466
Platelet (10^3^/μL)	248 (91–487)	239 (110–556)	0.763
Neutrophil (10^3^/μL)	5.6 (0.8–9.6)	5.3 (2.2–7.1)	0.717
Lymphocyte (10^3^/μL)	2 (0.3–7.8)	2.1 (0.5–6.3)	0.325
MPV (fL)	9.8 (6–14.4)	10.2 (5.9–16)	<0.001
Creatinine (mg/dL)	0.92 (0.47–1.9)	0.92 (0.34–1.9)	0.643
Urea (mg/dL)	16 (14–44)	16 (12–46)	0.526
C-reactive protein (mg/dL)	5.3 (3–27)	5 (3–35)	0.334
NLR	2.7 (0.35–4.8)	2.4 (0.19–5.9)	0.360
PMI	2,332.6 (968–4,378)	2,437.8 (1,104–5,112)	0.005
Number of grafts (n)	3 (1–7)	3 (1–6)	0.258
Total perfusion time (min)	85 (44–189)	88 (39–185)	0.407
Cross clamp time (min)	58 (12–130)	62 (11–133)	0.255

* Chi-square test,

‡ Mann-Whitney U test [data are expressed as median (minimum–maximum)].

ACE-I: angiotensin-converting enzyme inhibitor; ARB: angiotensin receptor blocker; BMI: body mass index; COPD: chronic obstructive pulmonary disease; SYNTAX: synergy between percutaneous coronary intervention with taxus and coronary artery bypass surgery; LVEF: left ventricular ejection fraction; PoAF: postoperative atrial fibrillation; PMI: platelet mass index; NLR: neutrophil-to-lymphocyte ratio.

The patients included in the study were divided into two groups according to the development of PoAF (Group 1: those who did not develop PoAF and Group 2: those who developed PoAF) (Table 2). There were 419 patients in Group 1 and their median age was 62 (38–89) years, and there were 203 patients in Group 2 and their median age was 69 (41–85) years (p<0.001).

**Table 2:** Demographic data and perioperative characteristics of the patients according to the development of postoperative atrial fibrillation.

Variables	Group 1 PoAF (-) (n=419)	Group 2 PoAF (+) (n=203)	p-value
Age (years)	62 (38–89)	69 (41–85)	<0.001‡
Male gender, n (%)	338 (80.7%)	157 (77.3%)	0.334*
Hypertension, n (%)	276 (65.9%)	152 (74.9%)	0.023*
Diabetes mellitus, n (%)	127 (30.3%)	63 (31%)	0.854*
Hyperlipidemia, n (%)	166 (39.6%)	89 (43.8%)	0.315*
Smoking n (%)	149 (35.6%)	83 (40.9%)	0.198*
COPD, n (%)	33 (%7.9)	37 (18.2%)	<0.001*
Cerebrovascular event, n (%)	34 (8.1%)	15 (7.4%)	0.753*
BMI (kg/m^2^)	26.9 (23–39.6)	27.1 (23–41.1)	0.298
LVEF (%)	50 (30–72)	50 (20–65)	0.032‡
SYNTAX score I	12 (8–35)	17 (7–41)	<0.001‡
White blood cell (10^3^/μL)	8.8 (3.8–10.6)	8.6 (4.4–9.6)	0.410
Hematocrit (%)	40.6 (25.5–55)	39.9 (23–52)	0.213
Platelet (10^3^/μL)	236 (91–550)	241 (99–556)	0.091
Neutrophil (10^3^/μL)	5.5 (1.7–9)	5.2 (0.8–9.6)	0.210
Lymphocyte (10^3^/μL)	2.1 (0.5–7.8)	1.9 (0.3–6.9)	0.091
MPV (fL)	10 (6–14.2)	10.3 (5.9–16)	0.165
Creatinine (mg/dL)	0.9 (0.34–1.9)	0.98 (0.45–1.9)	0.294
Urea (mg/dL)	18 (16–38)	16 (12–46)	0.391
C-reactive protein (mg/dL)	5 (3–235)	5.3 (3–115)	0.569
NLR	2.5 (0.19–52.41)	2.7 (0.36–34.8)	0.092
PMI	2,397 (991.9–4,680)	2,450.1 (968–5,112)	0.026
Number of grafts (n)	3 (1–7)	3 (1–6)	0.259
Total perfusion time (min)	88 (39–189)	90 (40–180)	0.416
Cross clamp time (min)	62 (11–130)	59 (20–133)	0.234

* Chi–square test,

‡ Mann Whitney U test [data are expressed as median (minimum–maximum)] ACE–I,

angiotensin-converting enzyme inhibitor; ARB: angiotensin receptor blocker; BMI: body mass index; COPD: chronic obstructive pulmonary disease; LVEF: left ventricular ejection fraction; SYNTAX: synergy between percutaneous coronary intervention with taxus and coronary artery bypass surgery; MPV: mean platelet volume; NLR: neutrophil-to-lymphocyte ratio; PMI: platelet mass index.

There was no difference between the groups in terms of gender, smoking, diabetes mellitus, hyperlipidemia rate, history of cerebrovascular accident, body mass index, white blood cell, hematocrit values, lymphocyte, neutrophil, MPV, urea, creatinine, and NLR values. In Group 2, the frequency of hypertension, COPD frequency, SYNTAX score I value, and PMI value were significantly higher, while the left ventricular ejection fraction (LVEF) value was significantly lower (p=0.023, p<0.001, p<0.001, p=0.026, and p=0.032, respectively) (Table 2).

Univariate and multivariate logistic regression analyses were performed to reveal the predictive factors for the development of PoAF (Table 3). In univariate analysis results, being over 70 years of age [odds ratio (OR): 2.845, 95% confidence interval (CI) 1.750–5.388, p<0.001], having hypertension (OR: 0.654 95%CI 0.358–0.845, p=0.028), having LVEF below 30% (OR: 1.959, 95%CI 1.360–3.070, p<0.001), having COPD (OR: 2.154, 95%CI 1.660–4.227, p<0.001), and having high SYNTAX score I (OR: 1.290, 95%CI 1.070–1.889, p<0.001) and PMI values (OR: 0.816, 95%CI 0.604–0.965, p=0.029) were found to be significantly correlated with PoAF. According to the results of multivariate analysis, being over 70 years old (OR: 1.934, 95%CI 1.489–2.995, p<0.001), having an LVEF below 30% (OR: 1.550, 95%CI 1.190–2.236, p=0.012), and having COPD (OR: 1.663, 95%CI 1.339–2.191, p<0.001) were found to be independent predictors in predicting the development of PoAF.

**Table 3:** Logistic regression analysis of risk factors for postoperative atrial fibrillation.

Variables	Univariate analyses			Multivariate analyses		
	p	Exp(B) Odds ratio	95% CI Lower–Upper	p	Exp(B) Odds ratio	95% CI Lower–Upper
Age> 70 years	<0.001	2.845	1.750–5.388	<0.001	1.934	1.489–2.995
Hypertension	0.028	0.654	0.358–0.845	0.321	0.794	0.471–1.183
LVEF< 30%	<0.001	1.959	1.360–3.070	0.012	1.550	1.190–2.236
COPD	<0.001	2.154	1.660–4.227	<0.001	1.663	1.339–2.191
SYNTAX Score I	<0.001	1.290	1.070–1.889	0.088	1.115	0.897–1.440
MPV	0.217	0.896	0.654–1.190	–	–	–
PMI	0.029	0.816	0.604–0.965	0.275	1.090	0.769–1.337
NLR	0.114	1.197	0.789–1.698	–	–	–

COPD: chronic obstructive pulmonary disease; LVEF: left ventricular ejection fraction; SYNTAX: synergy between percutaneous coronary intervention with taxus and coronary artery bypass surgery; MPV: mean platelet volume; PMI: platelet mass index; NLR: neutrophil-to-lymphocyte ratio.

Correlation analysis was performed between platelet mass index (PMI) and SYNTAX score I value. As a result of the analysis, a weakly significant correlation was detected between the two values (r=0.185, p<0.001).

## DISCUSSION

Coronary artery bypass surgery is performed with high success rates with the developing techniques today, and PoAF is an important problem that may occur after these operations. In this study, we investigated the risk factors for PoAF by dividing patients who underwent CABG according to the spring and autumn seasons. We did not detect any differences in PoAF rates between seasons in our study group. However, we found preoperative MPV and PMI values to be significantly higher in patients who operated in the autumn months. In our analysis to reveal PoAF risk factors, we revealed a significant relationship between PMI and PoAF. As a result of multivariate analysis, we determined that being over 70 years old, having an EF value below 30, and having COPD were independent predictors of PoAF.

It is known that there may be changes in hematological parameters depending on the seasons. For example, it has been shown that there are decreases in hematocrit values during the summer months. It has also been demonstrated that there are increases in platelet numbers and activation due to low air temperatures. Accordingly, it has been determined that coronary thromboembolism, cerebrovascular thrombosis, and peripheral thromboembolism increase in the winter months^6^. Another study showed that platelet counts were higher in the autumn months in the northern hemisphere than in the spring months^5^. We also found MPV and PMI values to be significantly higher in the autumn months in our study group. In a study that investigated PoAF status according to seasons, the most common PoAF rate was observed in the winter months. In this study, PoAF rates were found to be similar in spring and autumn, which is consistent with our study^7^.

Platelets have an important place in the pathogenesis of inflammation and thrombosis. Average platelet volume value has an important place in platelet activation. Large platelets secrete important mediators for coagulation, inflammation, thrombosis, and atherosclerosis. In this regard, it has been revealed that there may be a relationship between MPV and cardiovascular diseases^8^. The effects of platelet-related factors on PoAF have been investigated in various studies in the literature.

In a retrospective study conducted by Erdem et al., 208 patients who underwent elective isolated CABG surgery were included. PoAF developed in 38 (22%) of the patients, and as a result of multivariate analysis, MPV (OR: 2.564, p=0.005) and CRP (OR:1.055, p=0.05) values were shown to be independent predictors for PoAF^9^. In another study conducted by Şaşkın et al., 1,138 patients who underwent CABG surgery were included retrospectively. PoAF occurred in 294 patients (25.8%). In this study, it was concluded that there was a significant correlation between MPV values and PoAF^5^. In another study conducted by Özsin et al., 93 patients who underwent off-pump CABG surgery were included. In this study, MPV values were found to be higher in patients who developed PoAF, and no statistically significant difference was detected. In this study, only the age variable was shown to be an independent predictor for PoAF^10^. In our study, we found that MPV values were higher in patients who developed PoAF. However, there was no statistically significant difference between the groups.

Platelet mass index is a parameter obtained by multiplying the average platelet volume and platelet count and is an important indicator of platelet activity^11^. A study revealed a significant relationship between high MPV values and the presence of atherosclerosis in psoriasis patients^12^. In another study conducted by Korkmaz et al., the importance of PMI value in the treatment of premature retinopathy was investigated. At the end of the study, it was revealed that there was a significant decrease in PMI values in parallel with the improvement of retinopathy^13^. In a recent study by Guzelburc et al., the relationship between PMI and PoAF was investigated. A total of 848 consecutive patients who underwent CABG or valve surgery combined with CABG were included in the study. As a result of the analysis, the preoperatively calculated PMI value was shown to be an independent predictor for PoAF (OR: 1.01, p<0.01)^14^. In our study conducted on isolated CABG patients, although we did not find the PMI value as an independent predictor for the development of PoAF, we concluded that there was a significant positive correlation between the development of PoAF and the PMI value (OR: 0.816, p=0.029).

Hypertension is a factor that increases stress on the vascular wall. This situation triggers inflammation. Thus, the risk of PoAF increases in hypertensive patients^15^. In our study, we found that there was a significant correlation (OR: 0.654, p=0.028) between hypertension and PoAF. With advancing age, increasing collagen deposits in cardiac tissues cause fibrosis, which leads to conduction problems. In addition, mobilization problems and noncompliance with breathing exercises in the postoperative period in elderly patient groups increase the risk of atrial fibrillation^16^. Accordingly, in this study, we revealed that being over 70 years of age is a strong independent predictor of the development of PoAF (OR: 1.934, p<0.001).

Histopathological changes occur in the heart chambers due to reduced ejection fraction. Due to this situation, the risk of atrial fibrillation increases in patients with low ejection fraction^17^. In a meta-analysis conducted by Yamashita et al., low EF was shown to be an important predictor for PoAF^18^. In our study, we found that the EF value below 30% was an independent predictor for PoAF (OR: 1.550, p=0.012). Pulmonary arterial pressures and right ventricular functions are affected due to COPD^19^. For this reason, the risk of PoAF increases in COPD patients^20^. In our study, we revealed that there is a significant relationship between the presence of COPD and PoAF, which is consistent with the literature.

### Limitations

The most important limitation of this study is that it is a retrospective study. Additionally, this is a single-center study and the number of patients was limited.

## CONCLUSION

In this study, we first demonstrated that MPV and PMI values were higher in patients in the autumn months. However, we concluded that there was no difference between the two seasons in terms of PoAF rates. Additionally, for the first time in the literature, we showed that there is a significant relationship between PMI value and the development of PoAF in patients who underwent isolated CABG.
